# Whole-genome phylogenetic analysis of *Mycobacterium avium* complex from clinical respiratory samples

**DOI:** 10.1128/spectrum.01600-24

**Published:** 2025-01-10

**Authors:** Chew Ka Lip, Joelle Go, Nur Aisyah Binte Abu Bakar, Sophie Octavia, Raymond Tzer Pin Lin, Jeanette W. P. Teo

**Affiliations:** 1 Department of Laboratory Medicine, National University Hospital, Singapore, Singapore; 2 School of Biotechnology and Biomolecular Sciences, University of New South Wales 7800 , Sydney, Australia; 3 National Public Health Laboratory, National Centre for Infectious Diseases, Singapore, Singapore; 4 Department of Microbiology and Immunology, Yong Loo Lin School of Medicine, National University of Singapore, Singapore, Singapore; Beijing Institute of Genomics, Chinese Academy of Sciences, Beijing, China

**Keywords:** nontuberculous mycobacteria (NTM), *Mycobacterium avium*complex (MAC), whole-genome sequencing (WGS), phylogenetic analysis, antibiotic resistance, transmission dynamics, environmental sampling

## Abstract

The *Mycobacterium avium* complex (MAC) is a common causative agent causing nontuberculous mycobacterial (NTM) pulmonary disease worldwide. Whole-genome sequencing was performed on a total of 203 retrospective MAC isolates from respiratory specimens. Phylogenomic analysis identified eight subspecies and species. *M. avium* subspecies *hominissuis* (MAH) was the overwhelmingly dominant species (148/203, 72.9%). The other seven identified species were *M. intracellulare* subsp. *yongonense* (18/203, 8.9%), *M. intracellulare* subsp. *chimaera* (10/203, 4.9%), *M. colombiense* (11/203, 5.4%), *M. paraintracellulare* (6/203, 3%), *M. marseillense* (5/203, 2.5%), *M. intracellulare* (3/203, 1.5%), and *M. avium* subspecies *paratuberculosis* (2/203, 1%). Significant genetic clustering was observed among MAH isolates. Notably, a large cluster (<12 SNPs) of 76 MAH isolates bearing the same sequence type was observed. The presence of closely related isolates within hospital settings raises concerns about transmission routes with environmental sources potentially playing a significant role. Based on susceptibility breakpoints that are available for clarithromycin, amikacin, linezolid, and moxifloxacin, low rates of clarithromycin (0.5%, 1/203) and amikacin (1.5%, 3/203) phenotypic resistance were observed. While linezolid and moxifloxacin resistance were 25.6% (52/2030) and 46.3% (94/203), respectively. Drug resistance-associated loci were searched for mutations linked to phenotypic drug resistance. Of the entire cohort, only one isolate was found to have a A2059G 23S rRNA (*rrl*) gene mutation responsible for macrolide resistance.

**IMPORTANCE:**

*Mycobacterium avium* complex (MAC) infections are increasingly challenging to manage due to their complex species diversity and varied resistance patterns. This study underscores the genetic diversity within MAC, identifying at least eight species and subspecies among 203 clinical isolates, with *M. avium* subsp. *hominissuis* (MAH) being most prevalent at 72.9%. Notably, genetic clustering was observed within MAH and *M. intracellulare* subsp. *chimaera*, suggesting potential transmission routes within healthcare settings. Clarithromycin and amikacin resistance was found to be uncommon, aligning with the rarity of resistance-associated genetic mutations. These findings emphasize the need for enhanced infection control measures and routine susceptibility testing to tailor antibiotic therapies effectively.

## OBSERVATION

*Mycobacterium avium* complex (MAC) accounts for approximately 80% of nontuberculous mycobacteria (NTM) pulmonary disease (PD) in different geographic regions. In Singapore, *Mycobacterium abscessus* is the most prevalent, responsible for about 40% of NTM infections, while MAC accounts for about 15–26% of PD cases (1). Guidelines recommend treating MAC-PD patients with the combination of a macrolide, ethambutol, and rifamycin, with or without an injectable aminoglycoside, for at least 12 months after culture conversion (2). Treatment of MAC lung disease is lengthy (15–18 months).

MAC has traditionally been represented by two species: *M. avium* and *M. intracellulare*. Revisions to genome classification have added at least ten other species to the complex including *M. chimaera* (3). MAC can be ubiquitously isolated. While environmental sources of MAC are well-documented, healthcare-associated transmission of NTM has been less frequently studied and performed largely in the context of CF care centers (4, 5).

In this study, genomic analyses were conducted on 203 MAC clinical isolates from six different hospitals. The primary objectives were to investigate the molecular epidemiology, phylogenetic relationships, and resistance profiles of these local isolates.

MAC respiratory isolates originated from six different hospitals (referred to as Hospital A to F) from January 2020 to May 2021. The isolate contributions were as follows: Hospital C – 120, Hospital A – 73, Hospital D – 6, Hospital B – 2, and Hospitals E and F – 1 each. Only non-duplicate bacterial isolates were included in the analysis. Epidemiological data were limited to isolate collection date and the hospital where patients received care. Clinical MAC were cultured on Middlebrook 7H10 agar plates supplemented with 10% oleic acid-albumin-dextrose-catalase (OADC) at 37°C for up to 2 weeks. Drug susceptibility testing was performed using the Sensititre SLOMYCO2 Susceptibility Testing Plate (Thermo Fisher Scientific, MA USA) according to the manufacturer’s instructions. Breakpoints were interpreted according to Clinical and Laboratory Standards Institute (CLSI) breakpoints (6). MIC distribution for antimicrobials with no available breakpoints in presented in Supplementary data (Table S1).

DNA was extracted using the DNeasy Blood & Tissue Kits (QIAGEN, Hilden, Germany) and sequenced on Illumina’s HiSeq 2000 system. Genomes were assembled with SPAdes version 3.9.0 (7). BUSCO (8) was used to assess genome completeness. As all 203 isolates had completeness scores of >95%, they were included in the genomic analysis (Table S2). Multilocus sequence typing (MLST) was performed using the PubMLST scheme for *Mycobacteria* spp. (https://pubmlst.org/organisms/mycobacteria-spp). New MLST allelic profiles were arbitrarily assigned in this study as ‘ST-A’ to ‘ST-Q’ (Table S3). For species identification, average nucleotide identity (ANI) values were calculated using the Pyani package (https://github.com/widdowquinn/pyani) using reference MAC genomes in the GenBank database. Core genome alignments were generated using Snippy (v4.6.0) (https://github.com/tseemann/snippy). For intra-species phylogeny, *M. avium* subsp. *hominissuis* HP17 (GCA_002716905.4) and *M. intracellulare* subsp. *chimaera* ZUERICH-1 (GCA_002219265.1) were used as reference genomes. Isolates were considered genetically similar, and classified within a cluster if they exhibited a distance of ≤30 SNPs (5). Pairwise SNP distance was calculated from the recombination-free core genome alignments using snp-dists (https://github.com/tseemann/snp-dists). RAxML (9) was used to generate phylogenetic tree. The phylogenetic tree was visualized and annotated using iTOL (10). For the detection of polymorphisms in drug-resistance-associated genes, sequencing reads were mapped using Snippy v4.3.0 (https://github.com/tseemann/snippy).

**FIG 1:**
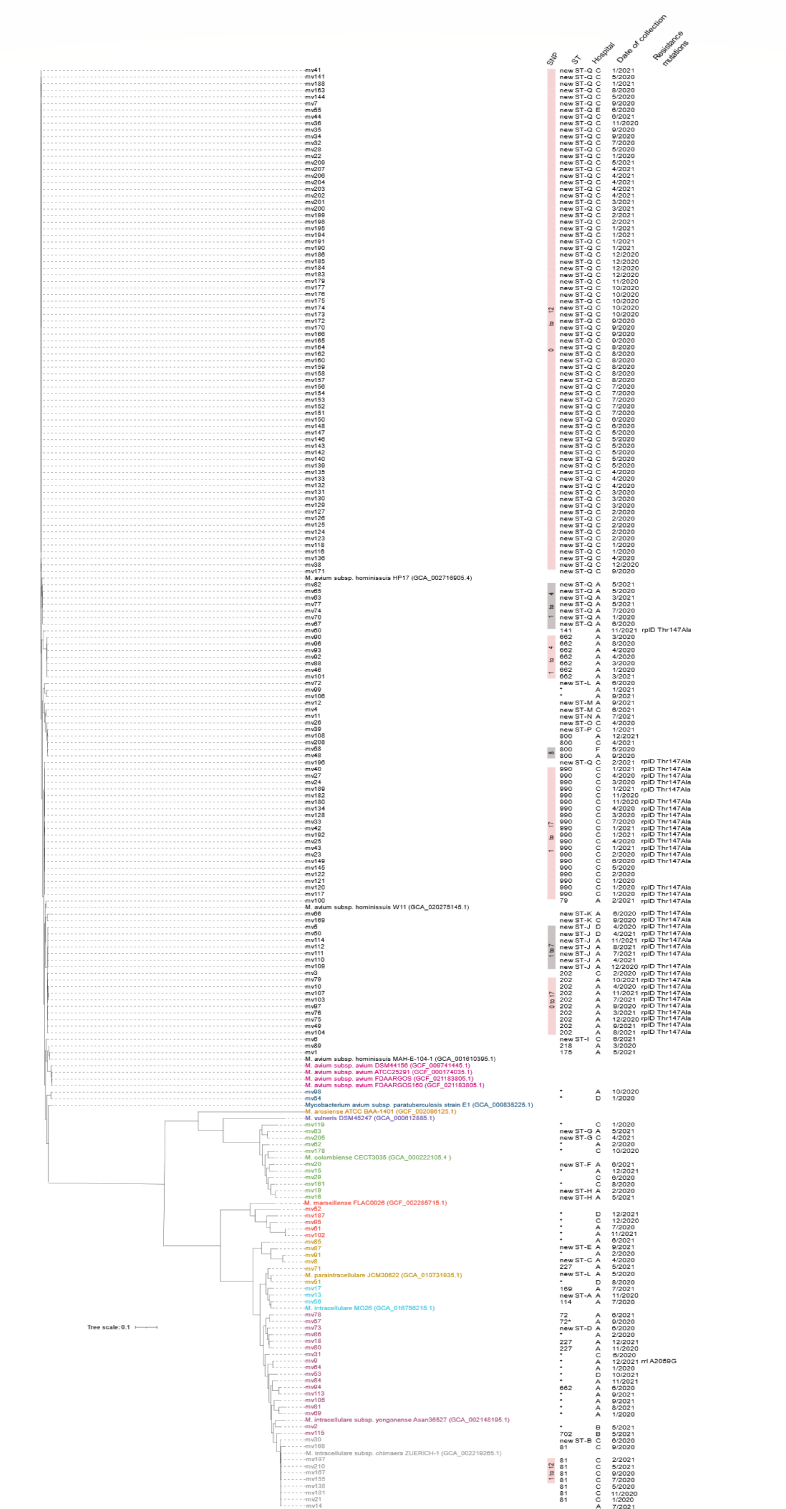
Core-SNP phylogenetic tree of 203 *Mycobacterium avium* complex (MAC) genomes analyzed in this study. Metadata displayed includes SNP counts, multi-locus sequence type (ST), hospital location, collection date (month/year), and identified resistance mutations. SNP counts are derived from core-SNPs obtained through intra-species genome alignments. Different species and subspecies are represented by distinct colors. New MLSTs were assigned arbitrarily in this study to indicate unique allelic Profiles. Profiles marked with an asterisk (*) indicate MLST profiles that have imperfect matches to existing alleles in the *Mycobacteria* spp. PubMLST typing database. GenBank accession numbers for reference assemblies are included in parentheses. All isolates sequenced in this study are labeled with the prefix "mv."

Phylogenetic analyses based on 10,865 bp core SNPs classified the 203 genomes into eight different species and subspecies, of which *M. avium* subspecies *hominissuis* (MAH) was the overwhelmingly dominant species (148/203, 72.9%). The other seven identified species were *M. intracellulare* subsp. *yongonense* (18/203, 8.9%), *M. intracellulare* subsp. *chimaera* (10/203, 4.9%), *M. colombiense* (11/203, 5.4%), *M. paraintracellulare* (6/203, 3%), *M. marseillense* (5/203, 2.5%), *M. intracellulare* (3/203, 1.5%), *M. avium* subspecies *paratuberculosis* (2/203, 1%), (Fig. 1). The species identification was also supported by ANI values which were typically >97% when compared to their species reference genome (data not shown).

MAH, one of four subspecies of *M. avium*, is clinically significant and causes disseminated disease in immunocompromised individuals (e.g., those with HIV) and PD in immunocompetent patients (3). Other subspecies include *M. avium* subsp. *avium* and *M. avium* subsp. *silvaticum*, both avian pathogens, and *M. avium* subsp. *paratuberculosis* (MAP), which causes John’s disease in ruminants and is linked to Crohn’s disease in humans. These other subspecies were not observed in our samples.

Overall, population structure shows isolate clustering amongst the MAH and *M. intracellulare* subsp. *chimaera* (Fig. 1). Noticeably there was a large cluster of 76 isolates (< 0 to 12SNPs) with same MLST (arbitrarily labeled as ST-Q) and from patients treated at the same hospital (Hospital C), with the exception of one isolate from Hospital E. We also noticed five other clusters of MAH with genomes possessing 0 to 17 SNPs. These clusters typically comprised isolates that also originated from patients who received treatment at the same hospital. (Fig. 1).

SNP threshold of ≤30 has been used to define cluster definition for *M. intracellulare* subsp. *chimaera* (5). Amongst the ten *M. intracellulare* subsp. *chimaera* isolates in our study four had ≤30 SNP differences, all were obtained from the same Hospital C (Fig. 1). In comparison, the distance between this cluster of isolates and the outbreak strain *M. intracellulare* subsp. *chimaera* ZUERICH-1 was >500 SNPs (Table S3), indicating that they were not related to ZUERICH-1. *M. intracellulare* subsp. *chimaera* has a long incubation period post-exposure, with a median of 17 months (range 3–72 months) (11). Therefore, it is conceivable that despite being isolated up to 8 months apart, the highly related strains point to a common source or a persistent transmission. Unfortunately, in this study, we were not able to obtain further epidemiological or clinical information as such we could not ascertain potential links among the genetic clusters.

Clarithromycin and amikacin susceptibility testing should be performed on MAC isolates prior to the initiation of treatment (6, 12). Where there is resistance to clarithromycin, CLSI (6) recommends drug susceptibility testing of moxifloxacin and linezolid, whereas the British Thoracic Society recommends testing a wider panel of antibiotics to guide treatment regimens (12). Clinical breakpoints have only been established for a few drugs, namely clarithromycin, amikacin, linezolid, and moxifloxacin (6).

Macrolides demonstrated excellent *in vitro* activity against MAC isolates, with 87.7% of the isolates demonstrating susceptibility (Table 1). Within our cohort, clarithromycin resistance was uncommon, identified in only 1.5% (3/203) of isolates (Table 1). Among these, just one *M. intracellulare* subsp. *yongonense* isolate harbored a A2059G mutation in the 23S rRNA gene (Fig. 1), while no mutations were detected in the 23S rRNA gene of the other resistant isolates. This low frequency of clarithromycin resistance aligns with other studies (13, 14), where macrolide resistance was similarly rare, ranging from 1.2% to under 3%. In contrast, a study from Southeast Asia (15) recorded nearly 50% clarithromycin resistance among their MAC isolates. High-level clarithromycin-resistant isolates typically possess point mutations at either position 2058 or 2059 of the 23S rRNA gene (Jamal, Maeda et al., 2000).

**TABLE 1:** Distribution of the susceptibility testing results of 203 *Mycobacterium avium* complex members versus clarithromycin, moxifloxacin, linezolid, and amikacin^*a*^^,*b*^

*Mycobacterium* spp.	MIC range (mg/L)	MIC50	MIC90	S (%)	I (%)	R (%)
Clarithromycin						
*M. intracellulare* subsp. *chimaera* *n* = 11	0.25–16	2	16	9/11 (81.8%)	2/11 (16.2%)	0
*M. intracellulare* subsp. yongonense *n* = 17	0.5–16	1	16	15/17 (88.2%)	1/17 (5.9%)	1/17 (5.9%)
*M. colombiense* *n* = 11	0.5–32	4	16	8/11 (72.7%)	1/11 (9.1%)	2/11 (18.2%)
*M. avium* subsp. *hominissuis* *n* = 148	0.25–16	4	8	134/148 (90.5%)	14/148 (9.5%)	0
*M. paratuberculosis* *n* = 2	8	8	8	2/2 (100%)	0	0
*M. marseillense* *n* = 5	8–16	8	16	3/5 (60%)	2/5 (40%)	0
*M. paraintracellulare* *n* = 6	2–16	8	8	5/6 (83.3%)	1/6 (16.7%)	0
*M. intracellulare* *n* = 3	8–16	8	16	2/3 (66.7%)	1/3 (33.3%)	0
All MAC *n* = 203	**0.25–16**	**4**	**16**	**178/203 (87.7%)**	**22/203 (10.8%)**	**3/203 (1.5%)**
Amikacin						
*M. intracellulare* subsp*. chimaera* *n* = 11	1–16	4	8	11/11(100%)	0	0
*M. intracellulare* subsp. yongonense *n* = 17	1–32	16	32	11/17(64.7%)	6/17 (35.3%)	0
*M. colombiense* *n* = 11	1–16	4	16	11/11 (100%)	0	0
*M. avium* subsp*. hominissuis* *n* = 148	1–32	4	16	142/148 (95.9%)	3/148 (2%)	0
*M. paratuberculosis* *n* = 2	8	8	8	2/2 (100%)	0	0
*M. marseillense* *n* = 5	2–8	4	8	5/5 (100%)	0	0
*M. paraintracellulare n* = 6	2–32	4	16	5/6 (83.3%)	1/6 (16.7%)	0
*M. intracellulare* *n* = 3	8–64	16	64	2/3 (66.7%)	0	1/3 (33.3%)
All MAC *n* = 203	**1–64**	**4**	**16**	**193/203 (95.1%)**	**9/203 (4.4%)**	**1/203 (0.5%)**
Linezolid						
*M. intracellulare* subsp. *chimaera* *n* = 11	8–>32	16	32	2/11 (18.2%)	5/11 (45.5%)	4/11 (36.4%)
*M. intracellulare* subsp. yongonense *n* = 17	8–>32	16	>32	5/17 (29.4%)	5/17 (29.4%)	7/17 (41.2%)
*M. colombiense* *n* = 11	2–>32	8	32	8/11 (72.7%)	1/11 (9%)	2/11 (18.2%)
*M. avium* subsp*. hominissuis n* = 148	2–>32	8	32	96/148(64.9%)	19/148 (12.8%)	33/148(22.3%)
*M. paratuberculosis n* = 2	32	32	32	0	0	2/2 (100%)
*M. marseillense* *n* = 5	2–32	8	16	3/5(60%)	1/5 (20%)	1/5(20%)
*M. paraintracellulare n* = 6	4–32	16	32	2/6 (33.3%)	1/6 (16.7%)	3/6 (50%)
*M. intracellulare* *n* = 3	4–16	8	16	2/3 (66.7%)	1/3 (33.3%)	0
All MAC *n* = 203	**1–32**	**8**	**32**	**118/203 (58.1%)**	**33/203 (16.3%)**	**52/203 (25.6%)**
Moxifloxacin						
*M. intracellulare* subsp. *chimaera* *n* = 11	1–4	4	4	1/11 (9.1%)	4/11 (36.3%)	6/11 (54.5%)
*M. intracellulare* subsp. *yongonense* *n* = 17	0.25–>4	2	>4	7/17 (41.2%)	6/17 (35.3%)	4/17 (23.5%)
*M. colombiense* *n* = 11	0.25–>4	2	>4	3/11 (27.3%)	4/11 (36.2%)	4/11 (36.2%)
*M. avium subsp. hominissuis* *n* = 148	0.12–>4	2	4	51/148 (34.5%)	24/148 (16.2%)	73/148 (49.3%)
*M. paratuberculosis* *n* = 2	4	4	4	0	0	2/2 (100%)
*M. marseillense* *n* = 5	0.5–>4	4	4	1/5(20%)	1/5 (20%)	3/5 (60%)
*M. paraintracellulare* *n* = 6	0.5–4	2	4	1/6 (16.7%)	3/6 (50%)	2/6 (33.3%)
*M. intracellulare* *n* = 3	0.5–2	2	2	1/3 (33.3%)	2/3(66.7%)	0
All MAC *n* = 203	**0.12–>4**	**2**	**4**	**65/203 (32%)**	**44/203 (21.7%)**	**94/203 (46.3%)**

^*a*^ n, number of isolates; S, susceptible; I, intermediate; R, resistant. MIC50 and the MIC90 were defined as the minimum concentration required to inhibit 50% and 90% of the isolates, respectively.

^*b*^ The breakpoints were interpreted according to CLSI (6) guidelines.

Amikacin resistance was uncommon, observed in only 0.5% (1/203) of isolates, with one isolate having an MIC of 64 mg/L (Table 1). Other studies have also observed that MAC isolates are mostly susceptible to aminoglycosides (13, 14). High-level amikacin resistance (MICs > 128 mg/L) in MAC isolates is associated with *rrs* (16S rRNA gene) mutations at A1408G, C1409T, and G1491T, with A1408G being the most dominant. In the isolate with an amikacin MIC of 64 mg/L, no mutations were detected, and as previously documented, *rrs* mutations do not always present themselves in isolates with elevated MICs (16).

Fluoroquinolone has been reported to have a therapeutic role in inducing culture conversion among patients with refractory MAC-PD, with persistent culture positivity after treatment initiation (17). Overall, linezolid resistance was 25.6% (52/203) (Fig. 1). This was comparable to the frequency observed in other studies. Reported linezolid resistance rates have shown a wide range of variability, of between 20% and 91.5% (13, 14)

Linezolid-resistant MAC isolates (MIC ≥32 mg/L) can carry mutations in several loci, namely, in *rrl* (23S rRNA gene) and in *rplC* and *rplD*. Missense mutations in *rrl* associated with the linezolid resistance have been identified as G2599A (in *M. avium*) and A2137T (in *M. intracellulare*) (18). In *M. tuberculosis*, the *rplC* T460C mutation confers linezolid resistance but this mutation has yet to be observed in linezolid-resistant MAC isolates (18, 19). We did not observe mutations in *rrl* and *rplC. rplD* substitutions Thr147Ala in *M. avium* and Arg148Lys in *M. intracellulare* could impart linezolid resistance (18). In our cohort, we observed that all 38 MAH isolates with the *rplD* Thr147Ala mutation displayed intermediate or resistant MICs (≥ 16 mg/L), suggesting that this mutation is associated with elevated resistance to linezolid (Fig. 1).

For all MAC isolates, the moxifloxacin resistance was 46.3%, (94/203). Similar to linezolid resistance, moxifloxacin resistances can range widely from as low as 3% (20) to higher resistance rates of 44.7% to 85.5% (14, 21). Mutations in the quinolone resistance-determining regions (QRDR) of *gyrA* and *gyrB* confer high levels of fluoroquinolone resistance. In our isolates with raised moxifloxacin MICs, mutations were not observed in the QRDR of *gyrA* and *gyrB*. Previous studies from Japan and South Korea also did not find any mutations in the *gyrA* and *gyrB* genes of moxifloxacin-resistant *M. avium* and *M. intracellulare* isolates (19). There appears to be no clear correlation between mutations of the DNA gyrase genes (*gyrA* and *gyrB*) and moxifloxacin resistance in MAC isolates, indicating that other mechanisms are involved in fluoroquinolone resistance.

This study’s findings offer important implications for antibiotic treatment strategies, infection control practices, and public health interventions. First, the low observed resistance rates to clarithromycin and amikacin among MAC isolates reaffirm the viability of these agents as primary options in treatment regimens for MAC PD. However, with linezolid and moxifloxacin resistance observed at higher frequencies (25.6% and 46.3%, respectively), our results suggest that susceptibility testing should be incorporated into treatment planning, particularly for patients unresponsive to initial therapy or those with prolonged treatment courses.

The genetic clustering, especially of MAH isolates in certain hospitals, underscores potential transmission pathways within healthcare settings. Although human-to-human transmission of MAC has not been documented (22), the clustering raises the possibility of indirect transmission, potentially via environmental sources in hospital settings (4). The ubiquitous nature of MAC, which can be commonly found in reservoirs such as municipal water, household faucets, and plumbing (23, 24), suggests that aerosolized water sources may serve as significant contributors to MAC pulmonary infections. Identifying and addressing these environmental reservoirs, especially in healthcare facilities, could inform targeted infection control measures, thus reducing transmission risks, particularly among immunocompromised patients who are highly susceptible to MAC infections.

## Data Availability

Raw sequence reads and assemblies in this study have been submitted to GenBank under project accession number PRJNA983112.

## SUPPLEMENTAL MATERIAL

Supplemental tables spectrum.01600-24-s0001.xlsx Tables S1 to S4.

ASM does not own the copyrights to Supplemental Material that may be linked to, or accessed through, an article. The authors have granted ASM a non-exclusive, world-wide license to publish the Supplemental Material files. Please contact the corresponding author directly for reuse.

## References

[1] Lim AYH, Chotirmall SH, Fok ETK, Verma A, De PP, Goh SK, Puah SH, Goh DEL, Abisheganaden JA. 2018. Profiling non-tuberculous mycobacteria in an Asian setting: characteristics and clinical outcomes of hospitalized patients in Singapore. BMC Pulm Med 18:85. doi:10.1186/s12890-018-0637-1 29788943 PMC5964916

[2] Daley CL, Iaccarino JM, Lange C, Cambau E, Wallace RJ Jr, Andrejak C, Böttger EC, Brozek J, Griffith DE, Guglielmetti L, Huitt GA, Knight SL, Leitman P, Marras TK, Olivier KN, Santin M, Stout JE, Tortoli E, van Ingen J, Wagner D, Winthrop KL. 2020. Treatment of nontuberculous mycobacterial pulmonary disease: an official ATS/ERS/ESCMID/IDSA clinical practice guideline. Eur Respir J 56:2000535. doi:10.1183/13993003.00535-2020 32636299 PMC8375621

[3] Busatto C, Vianna JS, da Silva LV Junior, Ramis IB, da Silva PEA. 2019. Mycobacterium avium: an overview. Tuberculosis (Edinb) 114:127–134. doi:10.1016/j.tube.2018.12.004 30711152

[4] AJv T, Ellis HC, Churchward CP, Kumar K, Ramadan N, Benson S, et al.. 2023. Mycobacterium avium complex genomics and transmission in a London hospital. Eur Respir J 61:2201237. doi:10.1183/13993003.01237-2022 36517182 PMC10116071

[5] Hasan NA, Davidson RM, Epperson LE, Kammlade SM, Beagle S, Levin AR, de Moura VC, Hunkins JJ, Weakly N, Sagel SD, Martiniano SL, Salfinger M, Daley CL, Nick JA, Strong M. 2021. Population genomics and inference of Mycobacterium avium complex clusters in cystic fibrosis care centers, United States. Emerg Infect Dis 27:2836–2846. doi:10.3201/eid2711.210124 34670648 PMC8544995

[6] CLSI. 2023. CLSI. Performance standards for susceptibility testing of mycobacteria, Nocardia spp., and other aerobic actinomycetes. 2nd ed. CLSI Supplement M24S. Clinical and Laboratory Standards Institute; CLSI standard document M24 Clinical and Laboratory Standards Institute, Wayne, PA.

[7] Bankevich A, Nurk S, Antipov D, Gurevich AA, Dvorkin M, Kulikov AS, Lesin VM, Nikolenko SI, Pham S, Prjibelski AD, Pyshkin AV, Sirotkin AV, Vyahhi N, Tesler G, Alekseyev MA, Pevzner PA. 2012. SPAdes: a new genome assembly algorithm and its applications to single-cell sequencing. J Comput Biol 19:455–477. doi:10.1089/cmb.2012.0021 22506599 PMC3342519

[8] Simão FA, Waterhouse RM, Ioannidis P, Kriventseva EV, Zdobnov EM. 2015. BUSCO: assessing genome assembly and annotation completeness with single-copy orthologs. Bioinformatics 31:3210–3212. doi:10.1093/bioinformatics/btv351 26059717

[9] Stamatakis A. 2014. RAxML version 8: a tool for phylogenetic analysis and post-analysis of large phylogenies. Bioinformatics 30:1312–1313. doi:10.1093/bioinformatics/btu033 24451623 PMC3998144

[10] Letunic I, Bork P. 2016. Interactive tree of life (iTOL) v3: an online tool for the display and annotation of phylogenetic and other trees. Nucleic Acids Res 44:W242–W245. doi:10.1093/nar/gkw290 27095192 PMC4987883

[11] Sommerstein R, Hasse B, Marschall J, Sax H, Genoni M, Schlegel M, Widmer AF, Swiss Chimaera Taskforce. 2018. Global health estimate of invasive Mycobacterium chimaera infections associated with heater–cooler devices in cardiac surgery. Emerg Infect Dis 24:576–578. doi:10.3201/eid2403.171554 29460746 PMC5823345

[12] Haworth CS, Banks J, Capstick T, Fisher AJ, Gorsuch T, Laurenson IF, Leitch A, Loebinger MR, Milburn HJ, Nightingale M, Ormerod P, Shingadia D, Smith D, Whitehead N, Wilson R, Floto RA. 2017. British thoracic society guideline for the management of non-tuberculous mycobacterial pulmonary disease (NTM-PD). BMJ Open Respir Res 4:e000242. doi:10.1136/bmjresp-2017-000242 PMC5663249 29449949

[13] Wetzstein N, Kohl TA, Andres S, Schultze TG, Geil A, Kim E, Biciusca T, Hügel C, Hogardt M, Lehn A, Vehreschild MJGT, Wolf T, Niemann S, Maurer FP, Wichelhaus TA. 2020. Comparative analysis of phenotypic and genotypic antibiotic susceptibility patterns in Mycobacterium avium complex. Int J Infect Dis 93:320–328. doi:10.1016/j.ijid.2020.02.059 32147539

[14] Maurer FP, Pohle P, Kernbach M, Sievert D, Hillemann D, Rupp J, Hombach M, Kranzer K. 2019. Differential drug susceptibility patterns of Mycobacterium chimaera and other members of the Mycobacterium avium-intracellulare complex. Clin Microbiol Infect 25:379. doi:10.1016/j.cmi.2018.06.010 29906595

[15] Sirichoat A, Kaewprasert O, Hinwan Y, Faksri K. 2023. Phenotypic drug-susceptibility profiles and genetic analysis based on whole-genome sequencing of Mycobacterium avium complex isolates in Thailand. PLoS One 18:e0294677. doi:10.1371/journal.pone.0294677 37992075 PMC10664917

[16] Kim S-Y, Kim DH, Moon SM, Song JY, Huh HJ, Lee NY, Shin SJ, Koh W-J, Jhun BW. 2021. Association between 16S rRNA gene mutations and susceptibility to amikacin in Mycobacterium avium Complex and Mycobacterium abscessus clinical isolates. Sci Rep 11:6108. doi:10.1038/s41598-021-85721-5 33731862 PMC7969740

[17] Koh W-J, Hong G, Kim S-Y, Jeong B-H, Park HY, Jeon K, Kwon OJ, Lee S-H, Kim CK, Shin SJ. 2013. Treatment of refractory Mycobacterium avium complex lung disease with a moxifloxacin-containing regimen. Antimicrob Agents Chemother 57:2281–2285. doi:10.1128/AAC.02281-12 23478956 PMC3632919

[18] Kim S-Y, Jhun BW, Moon SM, Jeon K, Kwon OJ, Huh HJ, Lee NY, Shin SJ, Daley CL, Koh W-J. 2019. Genetic mutations in linezolid-resistant Mycobacterium avium complex and Mycobacterium abscessus clinical isolates. Diagn Microbiol Infect Dis 94:38–40. doi:10.1016/j.diagmicrobio.2018.10.022 30581010

[19] Kim S-Y, Jhun BW, Moon SM, Shin SH, Jeon K, Kwon OJ, Yoo IY, Huh HJ, Ki C-S, Lee NY, Shin SJ, Daley CL, Suh GY, Koh W-J. 2018. Mutations in gyrA and gyrB in moxifloxacin-resistant Mycobacterium avium complex and Mycobacterium abscessus complex clinical isolates. Antimicrob Agents Chemother 62:e00527-18. doi:10.1128/AAC.00527-18 29914959 PMC6125518

[20] Fernandez-Pittol M, Batista-Arnau S, Román A, San Nicolás L, Oliver L, González-Moreno O, Martínez JA, Amaro-Rodríguez R, Soler N, Gené A, González-Cuevas A, Tudó G, Gonzalez-Martin J. 2022. Differences in drug-susceptibility patterns between Mycobacterium avium, Mycobacterium intracellulare, and Mycobacterium chimaera clinical Isolates: prospective 8.5-year analysis by three laboratories. Antibiotics (Basel) 12:64. doi:10.3390/antibiotics12010064 36671265 PMC9854862

[21] Chang C-L, Chen L-C, Yu C-J, Hsueh P-R, Chien J-Y. 2020. Different clinical features of patients with pulmonary disease caused by various Mycobacterium avium-intracellulare complex subspecies and antimicrobial susceptibility. Int J Infect Dis 98:33–40. doi:10.1016/j.ijid.2020.06.019 32534139

[22] Bryant JM, Grogono DM, Rodriguez-Rincon D, Everall I, Brown KP, Moreno P, Verma D, Hill E, Drijkoningen J, Gilligan P, et al.. 2016. Emergence and spread of a human-transmissible multidrug-resistant nontuberculous mycobacterium. Science 354:751–757. doi:10.1126/science.aaf8156 27846606 PMC5142603

[23] Lande L, Alexander D, Wallace R, Kwait R, Iakhiaeva E, Williams M, et al.. 2010. Mycobacterium avium in community and household water. Emerg Infect Dis J 25. doi:10.3201/eid2503.180336 PMC6390762 30789130

[24] Nishiuchi Y, Tamura A, Kitada S, Taguri T, Matsumoto S, Tateishi Y, Yoshimura M, Ozeki Y, Matsumura N, Ogura H, Maekura R. 2009. Mycobacterium avium complex organisms predominantly colonize in the bathtub inlets of patients’ bathrooms. Jpn J Infect Dis 62:182–186. 19468176

