# Erratum for Ka Lip et al., “Whole-genome phylogenetic analysis of *Mycobacterium avium* complex from clinical respiratory samples”

**DOI:** 10.1128/spectrum.03233-25

**Published:** 2025-11-14

**Authors:** Chew Ka Lip, Joelle Go, Nur Aisyah Binte Abu Bakar, Sophie Octavia, Raymond Tzer Pin Lin, Jeanette W. P. Teo

**Affiliations:** Beijing Institute of Genomics, Chinese Academy of Sciences, Beijing, China

## ERRATUM

Volume 13, no. 2, e01600-24, 2025, https://doi.org/10.1128/spectrum.01600-24. The editor’s name should appear as given in this Erratum.

